# A Bibliometric Analysis and Review of Supercritical Fluids for the Synthesis of Nanomaterials

**DOI:** 10.3390/nano11020336

**Published:** 2021-01-28

**Authors:** Wei Su, Hongshuo Zhang, Yi Xing, Xinyan Li, Jiaqing Wang, Changqing Cai

**Affiliations:** 1 School of Energy and Environmental Engineering, University of Science and Technology Beijing, Beijing 100083, China; suwei@ustb.edu.cn (W.S.); steve_zhs@126.com (H.Z.); lxy954745444@126.com (X.L.); b20200082@xs.ustb.edu.cn (J.W.); caichangqing@163.com (C.C.); 2 Key Laboratory of Knowledge Automation for Industrial Processes, Ministry of Education, Beijing 100083, China; 3 Beijing Key Laboratory of Resource-Oriented Treatment of Industrial Pollutants, University of Science and Technology Beijing, Beijing 100083, China

**Keywords:** supercritical, nanomaterials, bibliometrics

## Abstract

Since the 1990s, supercritical fluids for the synthesis of nanomaterials have been paid more and more attention by researchers and have gradually become one of the most important ways to prepare nanomaterials. In this study, literature data on “supercritical fluids for the synthesis of nanomaterials” from 1998 to 2020 were obtained from the Web of Science database, and the data were processed and analyzed by the bibliometric method combined with Microsoft office 2019, Origin 2018, VOSviewer, and other software, so as to obtain the research status and development trend of “supercritical fluids for the synthesis of nanomaterials”. The results show that since literature on “supercritical fluids for the synthesis of nanomaterials” appeared for the first time in 1998, the number of articles published every year has risen. In terms of this field, China has become the second-largest publishing country after the United States, and China and the United States display a lot of cooperation and exchanges in this field. “Supercritical CO_2_”, “supercritical water”, “supercritical antisolvent”, “surface modification”, and so on have become the research hotspots of “supercritical fluids for the synthesis of nanomaterials”.

## 1. Introduction

Since the discovery of the supercritical state in the 19th century, various studies have been carried out, and it has been applied and popularized in petrochemical and environmental protection [1], thermal power generation [2], food and drug industries [3,4], biomedical applications [5,6,7], material preparation [8,9], etc. This has gradually led to the formation of supercritical fluid extraction technology, supercritical fluid chromatography technology, supercritical dyeing technology, and supercritical drying technology, etc. Until the 1990s, “supercritical fluids” were applied in the field of materials science, which was inseparable from the in-depth study of nanoscience and nanotechnology.

Nanomaterials represent the most promising new materials of the 21st century. Because of their five significant effects, including their “volume effect”, “surface effect”, “quantum size”, “quantum tunnel”, and “dielectric confinement”, they are favored by researchers in the field of materials. Therefore, how to control the size, shape, and uniformity of nanoparticles and how to prepare nanomaterials are particularly important issues. 

“Supercritical fluids” refer to substances that exceed the critical temperature and pressure. When these factors exceed the critical point, the phase interface between the gas and liquid disappears and there is a “mixed gas” with liquid- and gas-related properties, such as a low viscosity, high density, good fluidity, mass and heat transfer characteristics, adjustable solvent selectivity, and the ability to dissolve insoluble substances under normal environmental conditions. At present, the most widely used solvents are supercritical carbon dioxide and supercritical water, which have been widely used in many fields because of their low price, environmental protection, and easy access to raw materials [10,11,12].

Water has a critical temperature (T = 374 °C) and pressure (P = 22.1 MPa). When the temperature and pressure of the system exceed the critical point, it is called supercritical water. This kind of gas-like liquid has many properties: (1) It has an extremely strong oxidation ability. The substance to be treated is placed in supercritical water and then dissolved with oxygen (which can be dissolved in a large amount). Its oxidation is stronger than that of potassium permanganate; (2) many substances can burn in it and give off flames; (3) many substances (such as oil) can be dissolved, and the volume will be greatly reduced when dissolved; (4) it can slowly dissolve and corrode almost all metals, even gold (similar to aqua regia); and (5) in supercritical water, chemicals will react quickly.

When the temperature is higher than the critical temperature (31.26 °C) and the pressure is higher than the critical pressure (7.29 MPa), the properties of carbon dioxide will change. Its density will be close to liquid, its viscosity will be close to gas, and its diffusion coefficient will be 100 times that of liquid, so it will have a strong dissolving ability [13]. It can dissolve many kinds of substances and then extract the effective components, exhibiting wide application prospects. Supercritical carbon dioxide is one of the most widely studied fluids, because it has the following characteristics: (1) The critical temperature of CO_2_ is 31.26 °C, the critical pressure is 7.29 MPa, and the critical conditions are easy to reach; (2) CO_2_ is inert in terms of chemical properties, colorless, odorless, nontoxic, and safe; and (3) CO_2_ has a low price and high purity and is easily accessible. Supercritical carbon dioxide (SC-CO_2_) has many unique properties, including an excellent mass transferability, gas diffusivity, and extremely low viscosity, so it is suitable for uniformly dispersing nanoparticles on support materials with a high surface energy [14].

Supercritical fluids used for the synthesis of nanomaterials have been developed on the basis of supercritical research. A large number of studies have found that when the temperature and pressure approach the critical point of the fluid, (1) the density of the fluid increases sharply, which affects the solubility of salt, and is conducive to supersaturation and particle nucleation; (2) the viscosity decreases and the reaction rate increases; and (3) the decrease of the dielectric constant and increase of the ion product greatly affect the hydrolysis of metal salts, resulting in supersaturation and particle nucleation [15]. These changes are beneficial to the preparation of nanomaterials. Based on these studies, supercritical fluids for the synthesis of nanomaterials have been developed. 

**Figure 1:**
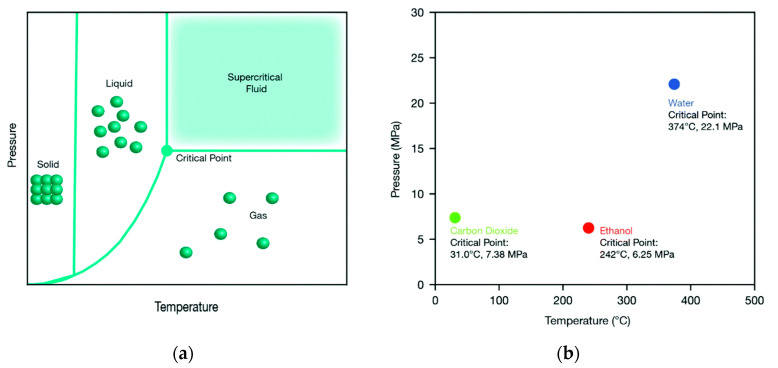
(**a**) Generic phase diagram highlighting the supercritical fluid region, and (**b**) critical temperatures and pressures for water, ethanol, and carbon dioxide, reproduced from [16], Copyright 2019, Royal Society of Chemistry.

Supercritical fluids employed for the synthesis of nanomaterials synthesize nanoparticles by using the unique properties of solvents in the supercritical state, and the morphology and particle size of nanoparticles are controlled by changing the synthesis conditions, such as the temperature, pressure, and solvent flow rate. As shown in Figure 1a,b, when the temperature and pressure of the medium exceed the critical point, the solvent is in a supercritical state. Since the supercritical state is only realized by a change of temperature and pressure, compared with other technologies for preparing nanomaterials, such as pulverization, gas condensation, precipitation, the hydrothermal method, the sol-gel method, etc., the system can be easily adjusted to the optimal preparation state, thus preparing the expected nanomaterials [16]. The fluid medium used for synthesizing nanomaterials by supercritical fluids is nontoxic and harmless, and the production of other substances and by-products is reduced in the reaction process. Moreover, the fluid medium can be recycled, so it is a kind of “green chemistry” [17]. Therefore, “supercritical fluids for the synthesis of nanomaterials” have the advantages of a high preparation efficiency, simple operation, greener environmental protection, and easier control of the shape and size of nanoparticles, so increasing numbers of researchers are paying attention to them internationally.

This research method based on bibliometrics has been widely used in scientific production and research trends in many disciplines and engineering fields. The purpose of this study is to use the traditional bibliometrics method to comprehensively process and analyze the data, including the author keywords, author countries, author institutions, cooperation relations among countries, etc. In this paper, the global data on “supercritical fluids for the synthesis of nanomaterials” from 1998 to 2020 (the first article in this field appeared in 1998) were sorted, the research trends were analyzed, and the future “supercritical fluids for the synthesis of nanomaterials” were prospected.

## 2. Data Sources and Methodology

The data in this paper were obtained from the Web of Science (http://apps.webofknowledge.com) core collection database. The seven keywords are composed of two parts: A and B. A stands for different expressions of nanomaterials, including “nanomaterials”, “nanotechnology”, “nanotechnology”, “nanoparticles”, or “NMs”, whilst B stands for different expressions of “supercritical”, including “supercritical” or “super-critical”. Statistical analysis was conducted for all related literature types, countries and affiliated institutions, literature authors, keywords, pages, subject classification, etc., in all databases from 1901 to 2020. The data collection date was 18 January 2021. In this econometric analysis, Microsoft office 2019 was used to process, retrieve, and analyze the data.

## 3. Results and Discussion

According to the statistical analysis, the SCI-Expanded network database includes 3815 publications related to supercritical fluids for the synthesis of nanomaterials for the period of time from 1 January 1998 to 31 December 2020. This paper systematically analyzes 3274 articles from 1998 to 2020.

### 3.1. Publication and Trends Analysis

**Figure 2:**
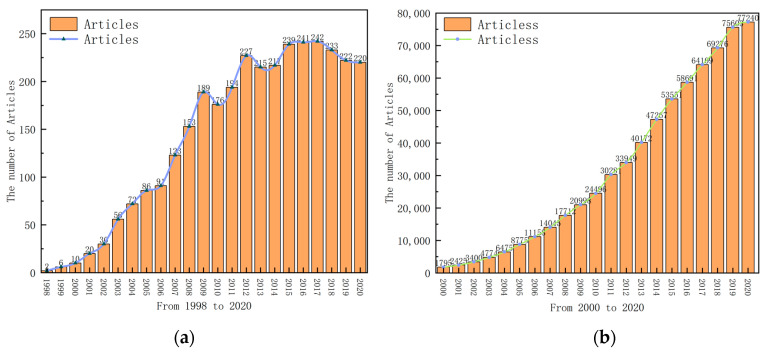
(**a**) The number of articles on “supercritical fluid for the synthesis of nanomaterials” and (**b**) the number of articles on “nanomaterials”.

**Figure 3:**
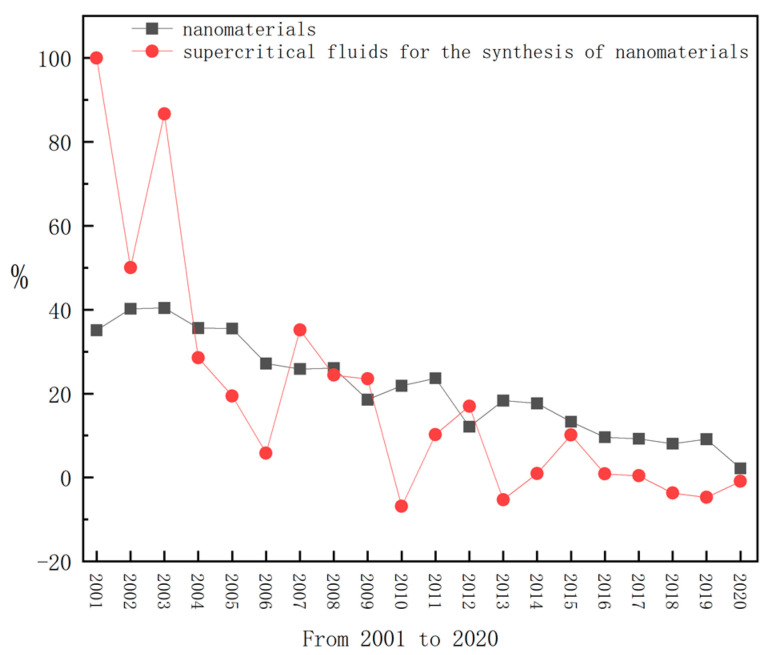
Percentage increase in the number of articles from 2001 to 2020.

The number of articles is an important index for measuring the development trend and current situation of a certain field. The number of articles on “nanomaterials” from 2000 to 2020 was obtained by the same method and compared with the number of articles on “supercritical fluid for the synthesis of nanomaterials”. Figure 2a shows the number of articles on “supercritical fluid for the synthesis of nanomaterials” from 1998 to 2020, whilst Figure 2b shows the number of articles on “nanomaterials” from 2000 to 2020. It can be seen from Figure 2a that reports on the supercritical preparation of nanomaterials began to appear in 1998, and there were few related articles from 1998 to 2002. Tour first reported the supercritical phenomenon in 1822. In the 1940s, scholars began to study the properties of supercritical fluids systematically. In 1959, Nobel Prize winner and physicist Richard P. Feynman first put forward the concept of “nanoparticles” in his speech. In 1984, Professor Gleiter of Germany put forward the concept of “nanomaterials”. Therefore, from 1998 to 2002, the technology of the supercritical preparation of nanomaterials attracted researchers’ attention. It can be seen from Figure 2b that the number of articles on “nanomaterials” is increasing and the base number is huge. The figure also shows that research on nanomaterials is becoming more and more popular among researchers. Figure 3 shows the changing trend of “supercritical fluid for the synthesis of nanomaterials” and the growth rate of “nanomaterials” from 2001 to 2020. It can be seen from Figure 3 that the growth rate of articles on “nanomaterials” has shown a downward trend in recent years, while the growth rate of “supercritical fluid for the synthesis of nanomaterials” has shown fluctuation. Additionally, except for a few years, the growth rate of articles on “supercritical fluid for the synthesis of nanomaterials” is similar to that of articles on “nanomaterials”. This shows that “supercritical fluid for the synthesis of nanomaterials” has always been the research focus and hot spot of researchers in the field of nanomaterials, and researchers are becoming increasingly interested in it.

### 3.2. Contribution of Country and Institution

To a certain extent, the number of papers published in a country can reflect its research level in this field. Through a statistical analysis of the addresses and affiliated institutions provided by the authors of periodical papers, the distribution of the number of published articles in each country was obtained. After reorganizing the classification, the statistics of articles from England, Scotland, Northern Ireland, and Wales belong to Britain, whilst “China” includes the Chinese mainland, Hongkong, Macau, and Taiwan. According to the quantitative analysis, 3274 articles were searched in the Web of Science database, which accurately provided the author’s institution and country. The number of single national papers and cooperative national papers was 2507 and 767, respectively, accounting for 76.57% and 23.43% of papers, respectively. From 1998 to 2020, a total of 52 countries (regions) published papers on supercritical fluids for the synthesis of nanomaterials. It can be seen from Table 1 that China was at the forefront of research in this field, and is now in second place, second only to the United States, with a small gap between the two countries. Furthermore, the number of papers published in this field in Japan, which ranks third, is far behind that of China. As of 2020, the United States has published 975 articles in this field, ranking first, accounting for 29.78% of papers; China ranks second, with 972 papers published, accounting for 29.69% of papers; and Japan ranks third, accounting for 11.79% of papers, being far behind the first two countries. The United States ranks first in terms of the number of articles published by CC, accounting for 55.54% of the sample. Additionally, China ranks first in terms of the number of articles published by SC and RC, accounting for 29.80% and 26.67% of papers, respectively. These data show that the United States and China have conducted a lot of research work in the field of “supercritical fluids for the synthesis of nanomaterials”, and the number of published papers for these countries ranks among the top in the world.

**Figure 4:**
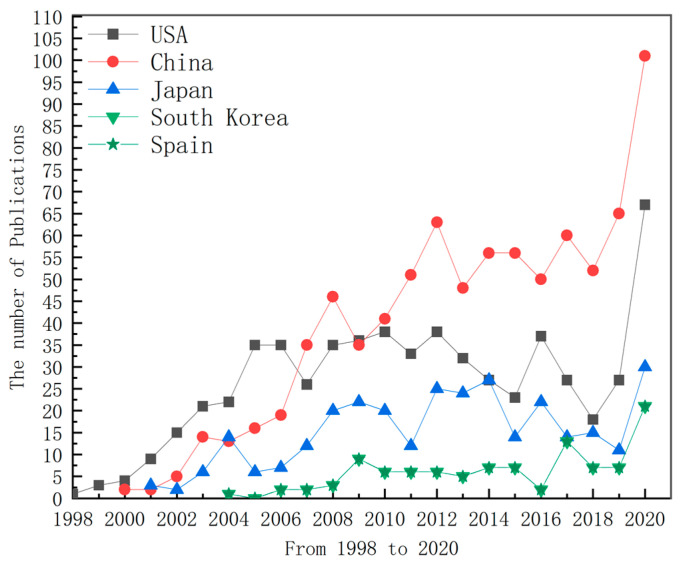
The national trend of the number of top five papers published from 1998 to 2020.

It can be seen from Figure 4 that the related research in this field first started in the United States. From 1998 to 2007, the number of articles published in the field of “supercritical fluids for the synthesis of nanomaterials” in the United States was higher than that in other countries. In 2007, China surpassed the United States for the first time in terms of the number of articles published in this field. After 2010, it surpassed the United States in an all-round way, ranking first up to now. In 2020, the United States and China published a large number of articles in this field, which indicates that research in this field has remained hot. This shows that since the 21st century, China has made great progress in scientific research, and the number of articles published has also significantly increased. 

To a certain extent, studying the number of cooperative publications between countries can reflect the communication between countries and whether the two countries are at the same level in this field. Table 2 shows the top ten countries that have cooperated with the United States and China in publishing articles. From the table, it can be seen that the United States and China have cooperated the most extensively with each other. The United States has cooperated with China in publishing 113 articles, accounting for 21.81% of the total published articles in the United States and 42.32% of the total published articles in China. It can be seen that the United States occupies a large proportion in Chinese cooperative publishing. At the same time, the articles published by the United States in cooperation with other countries are diverse. The second country to publish articles in cooperation with the United States is France, which has published 49 articles in cooperation with the United States, accounting for 9.46% of papers, and the third country is Spain, which has published 39 articles in cooperation with the United States, accounting for 7.53% of papers. The second country to publish articles in cooperation with China is Japan, which has published 30 articles in cooperation with China, accounting for 11.24% of papers, and the third country is Iran, which has published 13 articles in cooperation with China, accounting for 4.87% of papers.

**Figure 5:**
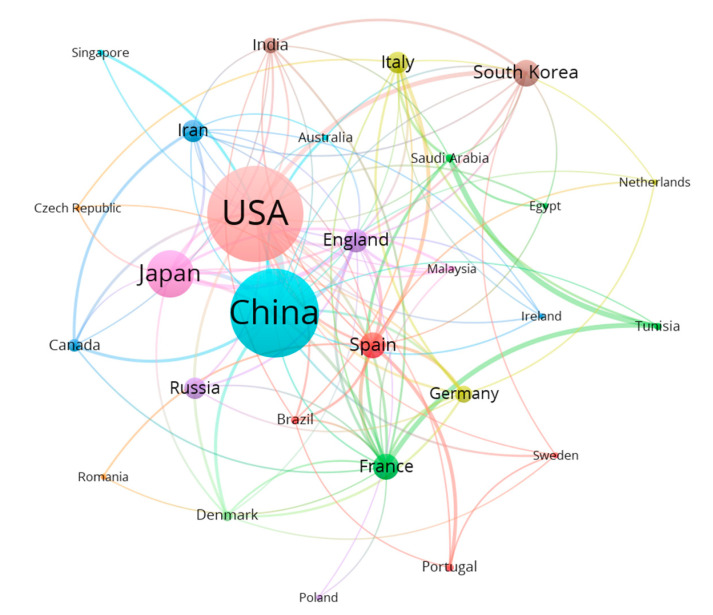
Country cluster map of cooperative published articles from 1998 to 2020.

We used VOSviewer software to analyze the countries that published articles during 1998–2020, and generated the cluster diagram shown in Figure 5. Only countries that have published twenty or more articles are shown in Figure 5, and only when fifteen or more articles have been jointly published by countries are they linked. From Figure 5, we can more intuitively see the comparison of the number of national publications and the closeness of cooperation and exchanges between countries from 1998 to 2020. Among them, China and the United States rank among the top two in terms of the number of articles published, and have the closest cooperation and exchanges.

Through a statistical analysis of the authors, the articles published by different research institutions were analyzed. According to the statistical analysis, the data of the 3274 analyzed papers in this article all include author institution information, and a total of 1866 research institutions have published 3274 articles. Among them, the total number of publications of independent institutions and cooperative institutions is 1418 (43.31%) and 1856 (56.69%), respectively. We selected 10 research institutions with the largest number of papers related to “supercritical fluids for the synthesis of nanomaterials” for analysis, as shown in Table 3. Among the top ten institutions that published articles, China, Italy, South Korea, Denmark, the USA, and France have one, and Russia and Japan each have two. The Chinese Academy of Sciences ranks first in the number of papers published in this field, with 167 papers (5.10%), followed by Tohoku University of Japan, with 118 papers (3.60%). Among them, the Chinese Academy of Sciences ranks first in terms of TI, SI, CI, FI, and RI indicators; Tohoku University ranks second in terms of TI, CI, FI, and RI, and ranks third in terms of SI. It can be seen from Table 1 that the total number of articles in the United States and China is similar, but it can be seen from Table 3 that the number of articles published by the Chinese Academy of Sciences is more than three times that for Idaho State University. This shows that the research institutes in this field are concentrated in China, mainly in the Chinese Academy of Sciences; however, there are many research institutes in this field in the United States, and their level is equivalent. The Chinese Academy of Sciences mainly pays attention to the preparation of electrode nanomaterials by a supercritical state [18], whilst Tohoku University pays attention to the formation and preparation of nanoparticles in a supercritical state [19].

### 3.3. Author Keywords Analysis

The keywords indicate the intention and purpose of the author’s research and summarize the key contents of the thesis. Therefore, a frequency analysis of keywords can reflect the hot topic and future development direction of a certain research field, which is beneficial for researchers who wish to quickly grasp the development trend of scientific research. In this paper, we used Microsoft Excel 2019 to sort the 3274 articles, and found that only 2242 articles gave keywords. This part summarizes and ranks the author keywords given in the 2242 articles. Because keywords are similar or have the same meaning, the keywords were classified one by one. The keywords with a similar or the same meaning were classified as one group, and finally, the 20 most frequently appearing keywords listed in Table 4 were obtained.

It can be seen from Table 4 that keywords such as “nanomaterials”, “supercritical carbon dioxide”, “supercritical fluid”, “supercritical water”, and “supercritical methanol” have a relatively high frequency. Among them, supercritical carbon dioxide, supercritical water, and supercritical methanol are all supercritical fluids. These three solvents represent three ways to prepare nanomaterials in a supercritical state. It can also be seen that the main types of “supercritical fluids for the synthesis of nanomaterials” are as follows: (1) Supercritical carbon dioxide for the synthesis of nanomaterials [20]. Rafael Camarillo and others successfully prepared nano-titanium dioxide by a supercritical carbon dioxide solvent, and achieved good results [21]; (2) supercritical water for the synthesis of nanomaterials. Chunbao Xu et al. [22] synthesized α-Fe_2_O_3_ nanoparticles in supercritical water with ferric nitrate as precursor solution, and deposited them on the surface and pores of activated carbon; and (3) supercritical organic solvent for the synthesis of nanomaterials. Nanometer barium titanate was prepared by J.F. Bocquet et al. [23] with supercritical isopropanol, and the particle size was about 10 nm. The sintering of particles and the dielectric properties of ceramics were tested.

**Figure 6:**
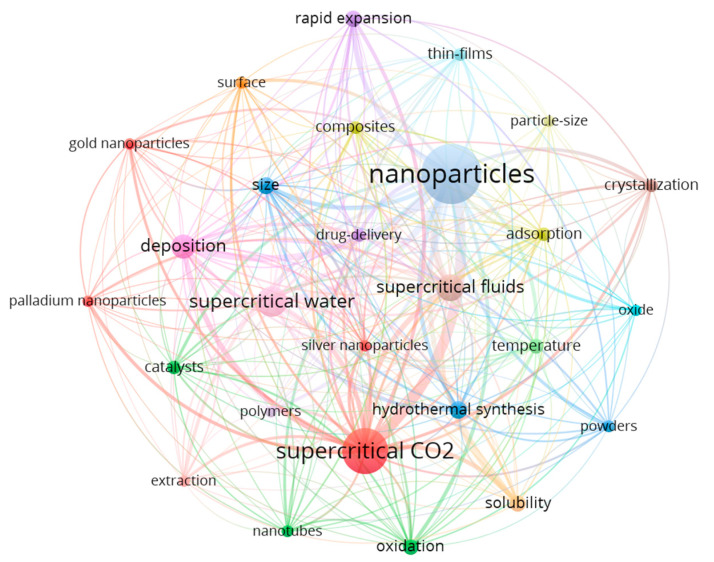
Author’s keyword cluster map for 1998 to 2020.

We used VOSviewer software to analyze the relevance of author keywords. Figure 6 shows the clustering diagram of author keywords. From the figure, we can clearly see the research frequency, research hot spots, and the relationship between keywords of “supercritical fluids for the synthesis of nanomaterials“. It can be seen from the figure that the research hotspots of “supercritical fluids for the synthesis of nanomaterials“ are “supercritical fluids”,” supercritical CO_2_”, “supercritical water”, “supercritical antisolvent”, “surface modification”, and so on. 

## 4. Hot Issues and Future Trends

According to the most common keywords counted in the author’s keyword analysis in Section 3.3, this chapter summarizes two aspects: Supercritical fluids for the synthesis of nanomaterials and future prospects. 

### 4.1. Supercritical Fluids for the Synthesis of Nanomaterials

Although the supercritical phenomenon was discovered in the 19th century, it was not until the 1990s that Professor Tadafumi Adschiri first synthesized metal oxide nanoparticles with supercritical water [24], and supercritical fluid gradually emerged in the preparation of nanomaterials. At present, “supercritical water for the synthesis of nanomaterials” and “supercritical carbon dioxide for the synthesis of nanomaterials” are the most widely studied. 

#### 4.1.1. Supercritical Water for the Synthesis of Nanomaterials

When water enters the supercritical state, the dielectric constant of water drops rapidly, and the dissolved metal salt precipitates rapidly at the same time. Because the precipitation process is extremely short (generally less than 0.1 s), the newly precipitated particles do not have enough time to combine with excess solute, which effectively inhibits the growth and agglomeration of particles. Most of the finally obtained particles will display the original particle size (critical particle size of about 5 nm) at the time of precipitation, thus producing nano-products [25,26]. The reaction process can be summarized as follows:

Hydrolysis reaction:

Mn^+^ + n H_2_O → M(OH)_n_ + n H^+^. (1)

Dehydration reaction:

M(OH)_n_ → MO_n/2_ + n/2 H_2_O. (2)

In the first step, metal ions dissolved in water are combined with OH^-^ ions in supercritical water to generate hydroxide for the hydrolysis reaction. Then, the newly generated hydroxide is rapidly dehydrated in supercritical water to generate nano metal oxide. For most ions, such as Ce, Ni, Zn, and Ti plasma, the above two reactions can be completed in the supercritical state. However, for some ions, such as La and Al plasma, it is difficult to conduct the second reaction because of their strong hydroxide stability and higher temperature required to open their chemical bonds. Generally, only hydroxide can be obtained [27].

**Figure 7:**
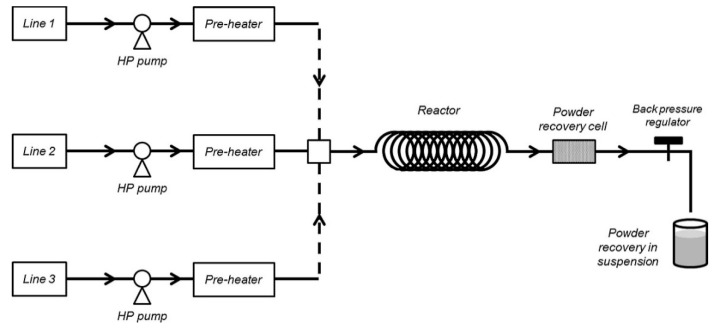
Flow chart of continuous supercritical water used for the synthesis of nanomaterials, reproduced from [24], Copyright 2014, Elsevier.

Figure 7 is a schematic flow chart of continuous supercritical water for the synthesis of nanomaterials. Line 1, Line 2, and Line 3 are three routes through which pure water, metal salt solution, and alkali solution can enter the supercritical reaction system. Pure water, metal salt solution, and alkali solution are fed by a high-pressure pump, and enter the preheating system to reach a near supercritical temperature. After passing through the mixer, they enter the supercritical reaction kettle to reach a supercritical state. The system pressure is regulated by a back pressure valve. The device is simple, flexible, and easy to operate and has a good stability. At present, the supercritical reaction system is widely used in the preparation of nanomaterials.

Gilles Philippot and Catherine Elissalde et al. [24] synthesized barium titanate-based nanomaterials with a core-shell structure by employing a supercritical state. In a few tens of seconds, barium titanate nanoparticles with good crystallization, a particle size of about 20 nm, and a narrow particle size distribution were produced. Yukiya Hakuta and Hiroshi Takashima et al. [28] synthesized perovskite-type metal oxide XTiO_3_ (X = Ca, Sr, Ba, Pb) nanoparticles in supercritical water at 400 °C and 30 MPa, and the order of particle size was Sr < Ca < Ba < Pb. Chunbao Xu and Amyn S et al. [22] synthesized α-Fe_2_O_3_ in a supercritical state and deposited it on the surface and pores of activated carbon particles. It was found that the dispersion of -Fe_2_O_3_ particles in activated carbon mainly depends on the soaking time in precursor solution at room temperature. The size and size distribution of particles are mainly affected by the concentration of precursors. Un Teng Lam and Raffaella Mammucari et al. [29] prepared hematite and magnetite nanomaterials by supercritical water synthesis, using supercritical water as a reaction medium. They believe that the synthesis process is rapid and simple, and the particle size and crystal formation can be controlled by operating conditions and changing the redox potential reflecting the environment. Mehrnoosh Atashfaraz and Mojtaba Shariaty-Niassar et al. [30] synthesized nano barium titanate particles under supercritical water (400 °C and 30 MPa) with barium hydroxide and titanium dioxide as precursors, and discussed and analyzed the effects of the solubility, temperature, and pH value on the synthesis of nanomaterials. Kunxu Zhu and Guoxin Hu et al. [31] realized the synthesis of a new template-free and organic titanate nanostructure with a controllable phase state and morphology by batch supercritical water treatment (400 °C) with hydrogen peroxide solution in sodium hydroxide solution. By changing the pH value of the reaction system, nanomaterials with different configurations can be prepared. The changes of the phase state and morphology of the product with temperature under subcritical conditions were studied. It was concluded that the formation of titanium dioxide nanostructures mainly follows the dissolution-nucleation-growth mechanism, which indicates that the supercritical temperature and pH value of sodium hydroxide solution are the decisive factors controlling the nucleation and growth process, phase, and morphology. Linda J. Cote and Amyn S. Teja [32] continuously synthesized Fe_2_O_3_ and Co_3_O_4_ nano-materials in supercritical water, and calculated and analyzed the effects of the temperature and cation solubility on the synthesis of nanoparticles by studying the thermodynamic model of the system.

#### 4.1.2. Supercritical CO_2_ for the Synthesis of Nanomaterials

For a long time, supercritical CO_2_ has been considered one of the promising substitutes for organic solvents, in order to realize the green chemical and industrial processes, and it is easy to apply for solvent separation and recovery [33,34]. In recent years, supercritical carbon dioxide has been widely used in the preparation of nanomaterials. Examples include (1) the synthesis and CO_2_-assisted synthesis of monodispersed metal NPs, (2) micelles dispersed in supercritical CO_2_ as ‘nano-reactors’ for the growth of metal NPs, (3) the template growth of robust metal nanostructures, and (4) the deposition and immobilization of metal NPs on porous supports as composites [35].

John P. Quigley and Kevin Herrington et al. [36] successfully synthesized and separated large-scale primary carbon nanotube bulk materials by supercritical carbon dioxide, and the average agglomerate size was less than 8 μm. Yuan Meng and Fenghua Su et al. [37], assisted by supercritical carbon dioxide, synthesized nickel nanoparticle point graphene oxide by chemical deposition. The lubricating performance of scandium nickel/gallium oxide composites synthesized by supercritical carbon dioxide was better than that of nano nickel, gallium oxide nanosheets, and nickel/gallium oxide composites without supercritical carbon dioxide. R. A. Dvorikova and L. N. Nikitin et al. [38] synthesized hyperbranched ferrocene polyphenyl magnetic nanomaterials based on supercritical carbon dioxide. Through SEM characterization, it was found that the size of nanoparticles formed in the polystyrene matrix containing ferrocene depends on the chemical structure of the catalyst used, and the mechanism of synthesizing this new magnetic nanomaterial was explored. Chien-I Wua and Jiann-Wen Huangand [39] successfully prepared nano-titanium dioxide powder by the supercritical fluid microemulsion system and supercritical drying method with titanium dioxide precursor titanium isopropoxide (TTIP) as raw material. Shancheng Yan et al. [40] successfully synthesized CdS semiconductor nanomaterials in supercritical carbon dioxide medium. The material had a uniform particle size distribution; its particle size ranged from 5 to 10 nm; and it exhibited good gas diffusion, a low viscosity, and a high-quality permeability. The photoelectric properties of CdS semiconductor nanomaterials were analyzed.

### 4.2. Future Prospects

Supercritical fluid has great advantages in the preparation of nanomaterials, and it is a promising direction in the preparation of nanomaterials. Because of its small particle size, uniform morphology, good dispersion, and simple reaction control conditions, it has attracted more and more scholars’ attention [41]. At present, a large number of experimental studies have been carried out in the direction of nanomaterials, such as those on Fe_2_O_3_ [22,29,42], TiO_2_ [31], CeO_2_ [19,43,44], and perovskite-type [24,26,30] and the corresponding nanomaterials have good properties. We believe that increasing numbers of scholars will be interested in supercritical fluids for the synthesis of nanomaterials in the future. We think future research should focus on how to achieve a stable synthesis of nanomaterials by controlling reaction conditions, so that they have a better dispersion and uniform morphology and particle size.

## 5. Conclusions

In this study, articles on “Supercritical Fluids for the Synthesis of Nanomaterials” published in the past 23 years were analyzed, and the related data were processed by the bibliometric method. The results show that, (1) since the article “supercritical fluids for the synthesis of nanomaterials” appeared for the first time in 1998, this field has been a research hotspot and focus in the field of nanomaterials, and researchers have grown increasingly interested in it; (2) the United States is the country with the largest number of articles in this field, and China and Japan are ranked second and third, respectively. Additionally, China and the United States have conducted a lot of cooperation and exchanges in this field; and (3) through the analysis of “author keywords”, it was found that the research hotspots of “supercritical fluids for the synthesis of nanomaterials” are “supercritical CO_2_”, “supercritical water”, “supercritical antisolvent”, “surface modification”, and so on. This reflects the research direction in this field. This paper also presents a literature review in the direction of “supercritical fluids for the synthesis of nanomaterials”. Supercritical water and supercritical carbon dioxide have great advantages in the preparation of nanomaterials. Many researchers have successfully synthesized a large number of nanomaterials and conducted in-depth research.

## Figures and Tables

**Table 1:** Top ten countries (regions) that published papers from 1998 to 2020.

Rank	Country	TC	TC R(%)	SC R(%)	CC R(%)	FC R(%)	RC R(%)
1	USA	975	1 (29.78)	2 (21.90)	1 (55.54)	2 (21.62)	2 (21.71)
2	China	972	2 (29.69)	1 (29.80)	2 (29.34)	1 (26.91)	1 (26.67)
3	Japan	386	3 (11.79)	3 (11.05)	4 (14.21)	3 (9.93)	3 (9.94)
4	South Korea	208	4 (6.35)	4 (5.82)	8 (8.08)	4 (5.65)	4 (5.69)
5	Spain	193	5 (5.89)	8 (3.31)	3 (14.34)	7 (4.31)	7 (4.34)
6	Iran	183	6 (5.59)	6 (4.67)	6 (8.60)	5 (4.73)	5 (4.62)
7	France	173	7 (5.28)	9 (3.11)	5 (12.39)	9 (3.15)	9 (3.36)
8	Russia	168	8 (5.13)	5 (4.83)	9 (6.13)	6 (4.49)	6 (4.50)
9	Italy	153	9 (4.67)	7 (3.59)	7 (8.21)	8 (3.73)	8 (3.79)
10	India	90	10 (2.75)	10 (2.15)	11 (4.69)	10 (2.05)	10 (2.02)

Note: TC is the total number of publications in a country/region; TC R(%) is the ratio of the number of publications in a country to the total number of publications; SC R(%) is the ratio of the number of publications in a single country to the total number of publications in all single countries; CC R(%) is the ratio of the number of publications in one partner country to the total number of publications in all partner countries; FC R (%) is the ratio of the number of publications in one country/region to the total number of publications in all countries/regions (this country is the country where the first author is located); and RC R (%) is the ratio of the number of publications in one country/region to the total number of publications in all countries/regions (this country/region is the country where the correspondent is located).

**Table 2:** The number of articles published in the top ten countries displaying cooperation between the United States and China from 1998 to 2020.

Rank	Country	USA	%	Rank	Country	China	%
1	China	113	21.81	1	USA	113	42.32
2	France	49	9.46	2	Japan	30	11.24
3	Spain	39	7.53	3	Iran	13	4.87
4	Japan	38	7.34	4	Canada	9	3.37
5	Saudi Arabia	34	6.56	5	Spain	9	3.37
6	South Korea	32	6.18	6	Sweden	7	2.62
7	Italy	26	5.02	7	Brazil	7	2.62
8	Turkey	21	4.05	8	Australia	6	2.25
9	Russia	15	2.90	9	South Korea	6	2.25
10	UK	13	2.51	10	India	6	2.25

**Table 3:** Top ten institutions that published papers from 1998 to 2020.

Rank	Institution	TI	TI R(%)	SI R(%)	CI R(%)	FI R(%)	RI R(%)
1	Chinese Academy of Sciences, China	167	1 (5.10)	1 (5.08)	1 (5.12)	1 (3.70)	1 (3.61)
2	Tohoku University, Japan	118	2 (3.60)	3 (3.10)	2 (3.99)	2 (2.63)	2 (2.66)
3	Russian Academy of Sciences, Russia	89	3 (2.72)	4 (1.97)	3 (3.29)	4 (2.08)	4 (2.02)
4	University of Salerno, Italy	86	4 (2.63)	2 (4.02)	11 (1.35)	3 (2.50)	3 (2.45)
5	National Institute of Advanced Industrial Science and Technology, Japan	66	5 (2.02)	10 (1.27)	5 (2.59)	5 (1.37)	5 (1.41)
6	Korea Advanced Institute of Science and Technology, South Korea	58	6 (1.77)	72 (0.28)	4 (2.91)	7 (1.13)	9 (0.83)
7	Moscow State University, Russia	53	7 (1.62)	22 (0.71)	6 (2.32)	14 (0.73)	13 (0.73)
8	Aarhus University, Denmark	49	8 (1.50)	6 (1.48)	10 (1.40)	6 (1.22)	6 (1.25)
9	University of Idaho, USA	46	9 (1.41)	6 (1.48)	11 (1.35)	8 (0.92)	7 (0.92)
10	University of Bordeaux, France	45	10 (1.37)	43 (0.42)	7 (2.10)	18 (0.61)	24 (0.55)

Note: TI is the number of total publications in an institution; TI R(%) is the ratio of the number of publications of an institution to the total number of publications; SI R(%) is the ratio of the number of publications of a single institution to the total number of publications of all single institutions; CI R(%) is the ratio of the total number of publications of a cooperative institution to the total number of publications of all cooperative institutions; FI R(%) is the ratio of the number of publications of one institution to the total number of publications of all institutions (where the institution is that of the first author); and RI R(%) is the ratio of the number of publications of one institution to the total number of publications of all institutions (the institution is the institution where the correspondent works).

**Table 4:** Top 20 keywords with the highest frequency from 1998 to 2020.

Rank	Author Keyword	98-19 TP	98-19 R(%)
1	nanoparticles	546	1 (24.35)
2	supercritical carbon dioxide	407	2 (18.15)
3	supercritical fluids	261	3 (11.64)
4	supercritical water	122	4 (5.44)
5	microparticles	74	5 (3.30)
6	supercritical antisolvent	61	6 (2.72)
7	aerogel	55	7 (2.45)
8	hydrothermal synthesis	52	8 (2.32)
9	sol-gel	52	8 (2.32)
10	supercritical	49	10 (2.19)
11	hydrogenation	43	11 (1.91)
12	palladium	36	12 (1.60)
13	carbon nanotubes	35	13 (1.56)
14	platinum	32	14 (1.42)
15	adsorption	31	15 (1.38)
16	ress	30	16 (1.34)
17	zinc oxide	30	16 (1.34)
18	surface modification	30	16 (1.34)
19	photocatalysis	30	16 (1.34)
20	supercritical methanol	26	20 (1.16)

## Data Availability

Data are available in the main text.
